# X-linked hyper-IgM syndrome presenting as severe *Pneumocystis* pneumonia in a 6-month-old infant: a case report

**DOI:** 10.3389/fimmu.2026.1929323

**Published:** 2026-08-26

**Authors:** Ying Guo, Xiaoli He, Lijun Zhang, Mengting Li, Lulu He, Guoyan Lu

**Affiliations:** 1 Department of Pediatrics, Ministry of Education Key Laboratory of Women and Children’s Diseases and Birth Defects, West China Second University Hospital, Sichuan University, Chengdu, Sichuan, China; 2 Department of Pediatrics, WCSUH-Tianfu Sichuan Provincial Children’s Hospital, Meishan, Sichuan, China

**Keywords:** CD40 ligand deficiency, infant, *Pneumocystis jirovecii* pneumonia (PJP), primary immunodeficiency, X-linked hyper-IgM syndrome (XHIGM)

## Abstract

**Background:**

X-linked hyper-immunoglobulin M (XHIGM) syndrome is a rare primary immunodeficiency caused by mutations in the CD40 ligand gene *(CD40LG)*, characterized by defective T-cell-dependent B-cell class-switch recombination. Patients typically present with recurrent infections in early childhood, but diagnosis is often delayed due to heterogeneous clinical manifestations and the fact that serum IgM levels may remain within the normal range in a significant proportion of patients. Here we report a case of XHIGM in an infant whose diagnostic journey began with recurrent lymphadenitis and culminated in life-threatening *Pneumocystis jirovecii* pneumonia (PJP).

**Case presentation:**

A 6-month-old male infant was admitted with severe respiratory distress and hypoxemia. He had recurrent axillary lymphadenitis at 1 and 2 months of age and significant failure to thrive. Chest CT showed bilateral consolidative and interstitial opacities. Immunological evaluation revealed markedly decreased IgA, normal IgM and IgG, and profound T-cell lymphopenia. Sputum metagenomic sequencing identified *Pneumocystis jirovecii*. Whole-genome sequencing identified a hemizygous likely pathogenic *CD40LG* mutation (NM_000074.3:c.520C>T, p.Q174*); his mother was a carrier. He was treated with mechanical ventilation, trimethoprim-sulfamethoxazole (TMP-SMX), micafungin, corticosteroids, and intravenous immunoglobulin (IVIG), and was discharged after 36 days.

**Conclusions:**

In infants with recurrent or opportunistic infections, persistently low IgA and declining T-cell counts—even when initial screening appears normal—should raise suspicion for underlying immunodeficiency and prompt genetic evaluation. Early aggressive management and evaluation for hematopoietic stem cell transplantation are essential to improve outcomes.

Serial immunological evaluation is essential in infants with recurrent or opportunistic infections, as persistently low IgA and declining T-cell counts—even when initial screening appears normal—should prompt genetic evaluation for underlying immunodeficiency. Early aggressive management and evaluation for hematopoietic stem cell transplantation are essential to improve outcomes.

## Introduction

X-linked hyper-immunoglobulin M syndrome (XHIGM) is a rare primary immunodeficiency disorder caused by mutations in the *CD40LG* gene, which encodes the CD40 ligand (CD40L) expressed on activated T cells. The disruption of CD40–CD40L interaction impairs T-cell-dependent B-cell class-switch recombination, leading to characteristically normal or elevated serum IgM, markedly decreased IgG and IgA, and concomitant T-cell dysfunction (1, 2). Clinically, patients typically present with recurrent sinopulmonary and opportunistic infections during infancy or early childhood (2, 3).

*Pneumocystis jirovecii* is an opportunistic fungus that causes severe pneumonia in immunocompromised hosts. In XHIGM patients, PJP is a severe complication and a major cause of early mortality, particularly in infants without prior diagnosis (4).

Here we report a case of XHIGM in a 6-month-old male infant who developed severe PJP following recurrent infections. This case illustrates the typical clinical trajectory of CD40L deficiency and emphasizes the need for prompt immunological evaluation followed by genetic testing in infants with early-onset, recurrent, or opportunistic infections.

## Case report

A 6-month-old male infant was admitted to our hospital with a 3-day history of progressive shortness of breath, cough, and lip cyanosis. His perinatal history was unremarkable. At 1 month of age, he developed a left axillary mass, which was diagnosed as lymphadenitis at a local hospital and resolved after antibiotic treatment. At 2 months of age, he was re-admitted for recurrent axillary lymphadenitis and respiratory syncytial virus (RSV) infection, from which he recovered with supportive care. Cellular immune function testing during those episodes reportedly showed no abnormalities. His parents were non-consanguineous. The patient has no male siblings. There is no known history of recurrent infections or early childhood deaths among maternal uncles or other male maternal relatives. His mother is healthy with no history of recurrent or severe infections.

On admission, the patient appeared lethargic with severe respiratory distress, including suprasternal, intercostal, and subcostal retractions, as well as head bobbing. Vital signs were: temperature 37.0 °C, respiratory rate 70 breaths/min, heart rate 168 beats/min, blood pressure 101/69 mmHg, and oxygen saturation (SpO_2_) 92% on facemask oxygen at 5 L/min. Anthropometric measurements showed a height of 65 cm (10th percentile) and weight of 6.3 kg (below the 3rd percentile), and head circumference of 42 cm (10th percentile), indicating significant growth failure. A visible scar was noted on the left upper arm from a previous axillary procedure, and a solitary soybean-sized enlarged lymph node was palpable in the left axilla. Chest auscultation revealed bilateral coarse crackles and wheezing. The liver was palpable 4 cm below the right costal margin, suggesting hepatomegaly.

Immunological evaluation revealed a striking temporal evolution of the patient’s immune profile (**Table 1**). At 2 months of age, T-cell subsets were quantitatively normal, but humoral immunity already showed reduced IgG (2.19 g/L) and IgA (0.06 g/L). By 6 months, T-cell subsets had declined dramatically to profound pan-T-cell lymphopenia (total T cells 1.34 × 10^9^/L, CD4^+^ 0.90 × 10^9^/L, CD8^+^ 0.42 × 10^9^/L), with near-undetectable IgA (< 0.20 g/L) and decreased complement C3 and C4. Of note, serum IgM remained within normal ranges at both time points. The 6-month IgG level (9.86 g/L) was measured after the patient had received intravenous immunoglobulin (IVIG, 5g) and therefore reflects exogenous replacement rather than endogenous synthesis. B-cell counts were normal despite a relative increase in percentage (63.0%).

**Table 1:** Longitudinal immunological findings at 2 and 6 months of age.

Parameter	2-month result	Reference range (2months)	6-month result	Reference range (6months)	Trend
T-cell subsets					
CD3^+^%	74.1%	48–75%	32.1%	50–77%	Markedly decreased
CD3^+^ (×10^9^/L)	4.75	2.30–6.50	1.34	2.40–6.90	Markedly decreased
CD4^+^%	61.7%	33–58%	21.4%	33–58%	Markedly decreased
CD4^+^ (×10^9^/L)	3.95	1.50–5.00	0.90	1.50–5.00	Markedly decreased
CD8^+^%	10.9%	11–25%	10.0%	13–26%	Normal→decreased
CD8^+^ (×10^9^/L)	0.70	0.50–1.60	0.42	0.60–2.20	Normal→decreased
CD4^+^/CD8^+^	5.7	1.5–2.0	2.1	1.5–2.0	Decreased
ALC					
(×10^9^/L)	6.24	2.40–9.50	7.47	2.40–9.50	slightly increased
B cells					
CD19^+^%	14.5%	14–39%	63.0%	13–35%	Relatively increased
CD19^+^ (×10^9^/L)	0.93	0.60–3.00	2.64	0.70–2.50	Normal
NK cells					
CD3^-^CD16^+^CD56^+^%	10.7%	2–14%	4.6%	2–13%	Normal
CD3^-^CD16^+^CD56^+^(×10^9^/L)	0.69	0.10–1.30	0.19	0.10–1.00	Normal
Immunoglobulins					
IgG (g/L)	2.19	2.75–12.3	9.86*	8.0–17.0	Low→normal*
IgA (g/L)	0.06	0.1–1.31	< 0.20	0.72–4.29	Persistently low
IgM (g/L)	0.35	0.21–1.92	0.82	0.6–2.6	Normal
Complement					
C3 (g/L)	1.12	0.74–1.76	0.58	0.78–2.10	Normal→decreased
C4 (g/L)	0.24	0.14–0.41	0.115	0.17–0.48	Normal→decreased

*The 6-month IgG level was measured after the patient had received IVIG (5 g). Reference ranges are age-adjusted according to established pediatric reference intervals used at our institution’s clinical laboratory.

Initial laboratory findings (**Table 2**) revealed leukocytosis with neutrophilia despite normal CRP and PCT—a pattern suggestive of non-bacterial or viral/opportunistic infection rather than bacterial sepsis.

**Table 2:** Laboratory parameters and respiratory support data before and after therapy.

Paremeter	Day 1 of MV	Day 3 of MV	Day 7 of MV	Prior to weaning
WBC (10^9/L)	15.79	23.38	13.22	12.71
ANC (10^9/L)	6.76	6.06	4.06	3.01
ALC (10^9/L)	7.47	14.93	7.72	7.46
CRP (mg/L)	<0.20	1.35	0.27	0.08
PCT(ng/ml)	0.037	-	-	0.02
pH	7.349	7.421	7.408	7.427
PaO_2_ (mmHg)	55	107	145	128
FiO_2_(%)	70	70	50	35
PaO_2_/FiO_2_	78	152	290	365.7
Lac (mmol/L)	3.29	0.79	–	–

MV, mechanical ventilation; WBC, white blood cell; ANC, absolute neutrophil count; ALC, absolute lymphocyte count; CRP, C-reactive protein; PCT, procalcitonin; FiO_2_, fraction of inspired oxygen; Lac, lactate.

**Figure 1:**
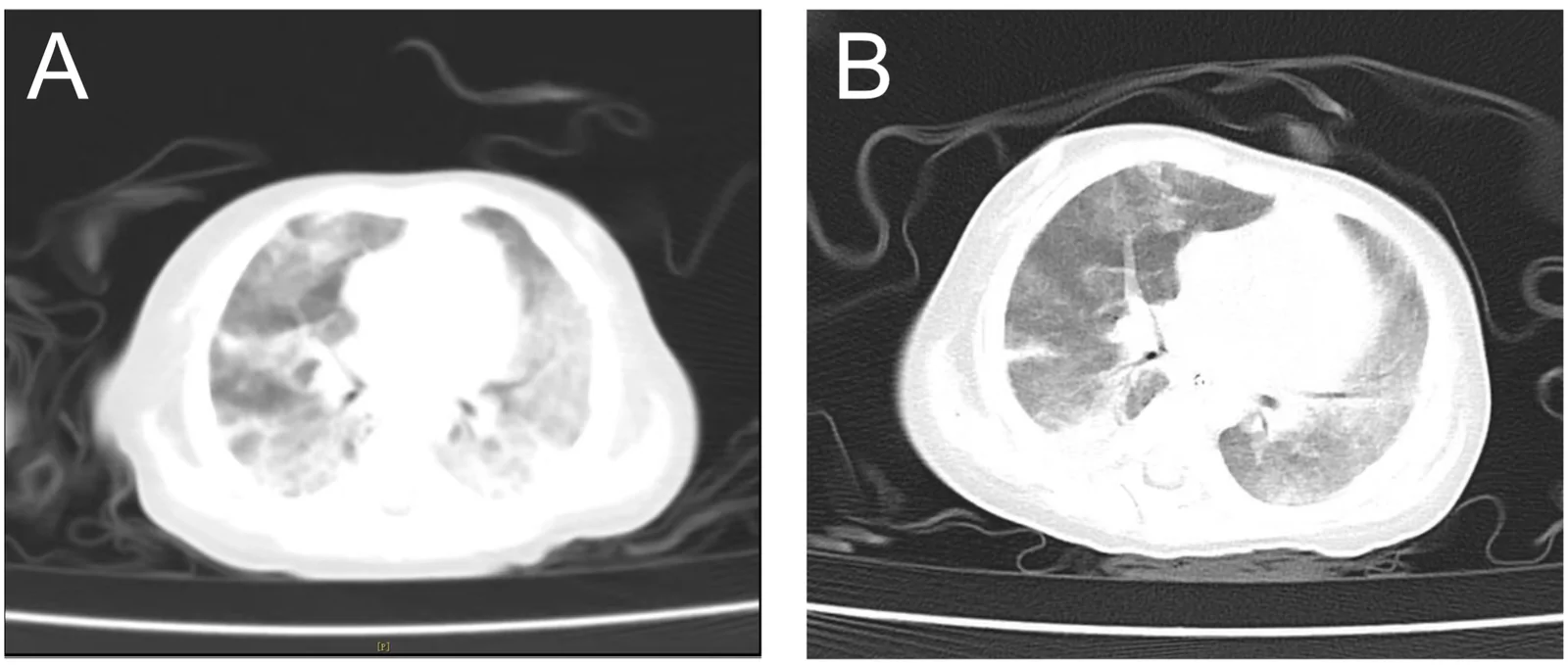
Imaging manifestations. **(A)** Chest CT on admission showing bilateral scattered ill-defined patchy, linear, reticular, and consolidative opacities, predominantly in the perihilar and lower lung zones, compatible with PJP in the appropriate clinical context. **(B)** Chest CT after 10 days of treatment showing significant interval improvement, with marked resolution of bilateral consolidative and reticular opacities, although scattered residual ground-glass opacities and linear densities persist in the perihilar and lower lung zones. high signal intensity.

Microbiological workup included respiratory pathogen nucleic acid testing, which was positive for human rhinovirus and RSV. Sputum culture grew *Staphylococcus lentus*. Metagenomic next-generation sequencing (mNGS) of induced sputum identified Pneumocystis jirovecii with 34,002 reads. Bronchoalveolar lavage was not performed due to the patient’s critical respiratory condition. Chest CT demonstrated bilateral scattered consolidative and interstitial opacities, with patchy ground-glass and reticular changes, as well as partial enlargement and calcification of the left axillary lymph nodes (**Figure 1**). Testing for *Mycobacterium tuberculosis* and HIV was negative. The diagnosis of active PJP was supported by the high mNGS read count, characteristic imaging findings, severe hypoxemic respiratory failure, and the patient’s underlying T-cell immunodeficiency.

### Treatment and hospital course

The patient required invasive mechanical ventilation for severe respiratory failure. Empirical antimicrobial therapy was initiated with vancomycin (15 mg/kg q6h), meropenem (40 mg/kg q8h), trimethoprim-sulfamethoxazole (TMP-SMX, 25 mg/kg q6h), and micafungin (5 mg/kg qd) as adjunctive therapy. Given the severity of hypoxemia, methylprednisolone was administered at 2 mg/kg/day for 5 days, then tapered sequentially to 1 mg/kg/day for 5 days, and subsequently to 0.5 mg/kg/day for another 5 days (total course of 15 days) to mitigate the excessive pulmonary inflammatory response associated with PJP (5). Intravenous immunoglobulin (IVIG, 1 g/kg) was empirically infused early in the course, prior to the definitive genetic diagnosis, in view of the clinical suspicion of underlying immunodeficiency.

Serial laboratory parameters and respiratory support data during the clinical course are summarized in **Table 2**. The patient’s oxygenation status improved progressively, with the PaO_2_/FiO_2_ ratio rising from 78 on day 1 of mechanical ventilation to 365.7 prior to weaning. The white blood cell count and neutrophil count peaked on day 3 and then gradually declined, while C-reactive protein remained low throughout, consistent with the initial pattern of non-bacterial infection. These trends reflected a favorable response to antimicrobial therapy and supportive care.

**Figure 2:**
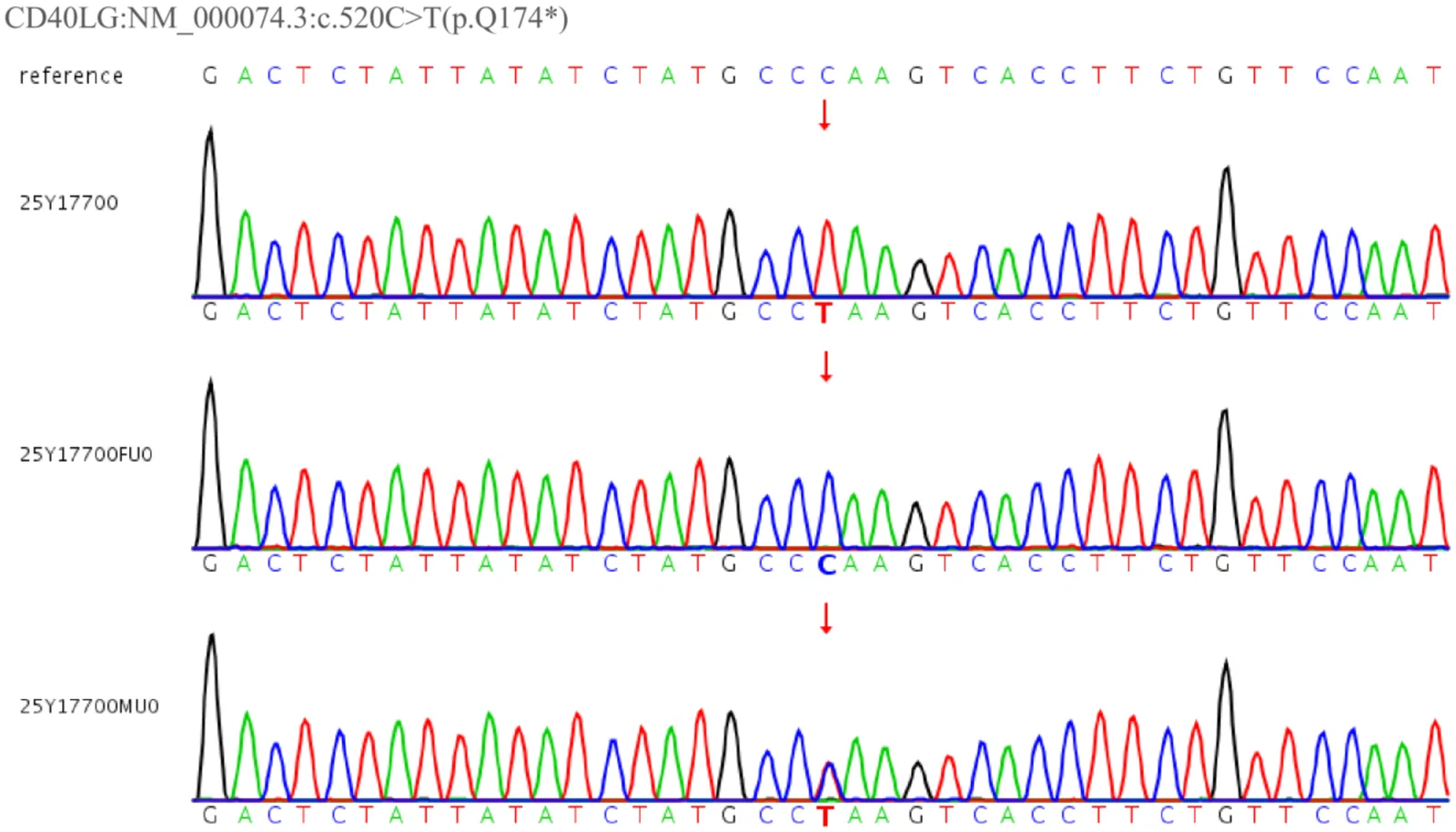
Sanger sequencing confirmation of the *CD40LG* mutation. The proband (25Y17700) carries a hemizygous c.520C>T (p.Q174*) mutation in the *CD40LG* gene. The mother (25Y17700MU0) is a heterozygous carrier, while the father (25Y17700FU0) is wild-type. The arrow indicates the mutation site.

Given the patient’s poor growth, recurrent infections, hepatomegaly, T-cell lymphopenia, and PJP infection, genetic testing was pursued. Whole-genome sequencing identified a hemizygous nonsense mutation in the *CD40LG* (NM_000074.3:c.520C>T, p.Q174*). According to the American College of Medical Genetics and Genomics (ACMG)/Association for Molecular Pathology (AMP) guidelines (6), this variant was classified as likely pathogenic (PVS1_Strong + PS4_Supporting + PM2_Supporting), consistent with the diagnosis of X-linked hyper-IgM syndrome (Online Mendelian Inheritance in Man (OMIM) #308230) (**Figure 2**). His mother was found to be a heterozygous carrier, while his father carried no mutation, confirming maternal inheritance.

After 36 days of combined intensive therapy, the patient was weaned from mechanical ventilation and discharged in stable condition. The patient has been receiving regular IVIG replacement therapy (500 mg/kg every 4 weeks) and continuous PJP prophylaxis with TMP-SMX. He is currently oxygen-independent and breathing room air. At 10 months of age (4 months post-discharge) the patient is scheduled for HLA-haploidentical related hematopoietic stem cell transplantation from his father.

## Discussion

This case report describes a 6-month-old male infant with XHIGM who presented with severe PJP following a history of recurrent axillary lymphadenitis. XHIGM is classified as a combined immunodeficiency within the international nomenclature of inborn errors of immunity (IEI) established by the International Union of Immunological Societies (IUIS) Expert Committee (7, 8). Several clinical features of this case merit further discussion.

Early recognition and diagnostic approach. Early recognition of primary immunodeficiency in infants is critical but challenging. The Jeffrey Modell Foundation has proposed 10 warning signs of Primary Immunodeficiency (PI), including recurrent infections, failure to thrive, need for intravenous antibiotics, and family history of PI (9) Our patient exhibited at least three of these signs (failure to thrive, recurrent lymphadenitis, and subsequent PJP), which should have prompted earlier immunological evaluation and genetic testing. This approach aligns with recent guidelines recommending rapid genetic testing for critically ill infants with suspected inborn errors of immunity to enable timely diagnosis and guide management (10). However, a normal serum IgM level—reported in approximately 50% of XHIGM cases (11) —can be falsely reassuring and delay diagnosis, as occurred in our patient. We proceeded directly to genetic testing without prolonged diagnostic uncertainty, and the identification of a likely pathogenic CD40LG mutation confirmed XHIGM. A key lesson is that early genetic testing should be pursued when clinical and immunological findings suggest immunodeficiency.

The differential diagnosis of opportunistic infections in infancy includes severe combined immunodeficiency (SCID) (12), DOCK8 deficiency (13), GATA2 deficiency (14, 15), and XHIGM (3). SCID was considered less likely given the presence of B cells and the initial normal T-cell counts at 2 months. DOCK8 deficiency typically presents with cutaneous viral infections (molluscum, HSV) and elevated IgE, which were absent. GATA2 deficiency usually manifests later with monocytopenia and mycobacterial infection syndrome. The combination of normal IgM, low IgA, T-cell lymphopenia, and PJP in a male infant was most consistent with XHIGM, subsequently confirmed by genetic testing.

A particularly instructive aspect of this case is the temporal evolution of the patient’s immune profile. At 2 months of age, T-cell subsets were quantitatively normal, but IgG (2.19 g/L) and IgA (0.06 g/L) were already below normal limits—findings initially attributed to physiological hypogammaglobulinemia of infancy. By 6 months, however, T-cell subsets had declined dramatically (total T cells from 4.75 to 1.34 × 10^9^/L), IgA became undetectable (< 0.20 g/L), and complement C3/C4 decreased significantly. This temporal pattern suggests possible evolution of T-cell lymphopenia (16); however, this observation is based on only two time points, and alternative explanations include severe acute PJP, viral coinfection, critical illness, and lymphocyte redistribution. Notably, the 6-month evaluation was performed prior to corticosteroid administration, partially mitigating this confounder. The concurrent decrease in complement C3 and C4 may reflect consumption during severe PJP infection, although this remains speculative without direct measures of complement activation. We emphasize that these observations are limited to a single patient, and longitudinal studies with additional time points are needed to determine whether T-cell deficiency in XHIGM is truly progressive.

### Genetic findings and genotype–phenotype correlation

The c.520C>T (p.Q174*) mutation identified in our patient is a nonsense variant located in exon 5 of the *CD40LG*, which results in premature termination of *the CD40L* protein and disruption of the last 88 amino acids. In silico analyses predict a deleterious effect on protein function, and the variant is absent from population databases (1000 Genomes, gnomAD), consistent with a rare disease-causing allele. This mutation has been previously reported in at least two independent cohorts of XHIGM patients (17, 18) and is classified as likely pathogenic according to ACMG/AMP criteria (PVS1_Strong + PS4_Supporting + PM2_Supporting). In the cohort reported by Lee et al., patients carrying this nonsense variant presented with recurrent sinopulmonary and opportunistic infections including PJP, consistent with the phenotype observed in our patient.

It should be acknowledged that functional confirmation of *CD40L* deficiency through flow cytometric assessment of *CD40L* surface expression on activated T cells was not performed in this patient. The diagnosis was established during an acute critical illness when functional immunological assays were not feasible. While the identification of a nonsense mutation (p.Q174*) predicted to result in premature protein termination, combined with the consistent clinical phenotype and maternal carrier status, provides strong genetic evidence for the diagnosis, functional assays such as *CD40L* expression analysis would have further strengthened the conclusion. This represents a limitation of the present report.

### Management considerations

The management of severe PJP in infants with XHIGM is challenging. Our patient received TMP-SMX combined with micafungin, along with systemic corticosteroids to mitigate pulmonary inflammation.

The use of micafungin as adjunctive therapy warrants comment. Although TMP-SMX remains the standard of care for PJP, adjunctive echinocandin therapy has been reported to improve outcomes in severe PJP in non-HIV immunocompromised patients, supported by a meta-analysis demonstrating reduced mortality with combination therapy (19) and a comprehensive review acknowledging echinocandins as a potential adjunctive option (20).In our patient, the decision was based on severe hypoxemic respiratory failure and the need for maximal coverage while awaiting definitive confirmation.

Although corticosteroids are well-established for PJP with moderate-to-severe hypoxemia in HIV-infected patients (5), their role in non-HIV immunocompromised children is less defined; our decision was based on severe respiratory failure and the need to reduce immune-mediated lung injury. Given the strong clinical suspicion of immunodeficiency, IVIG was administered empirically before genetic confirmation, and regular replacement therapy is planned post-discharge to reduce infection frequency (3, 21). Hematopoietic stem cell transplantation (HSCT) remains the only curative option (22, 23), and our patient is now listed for transplantation.

### Educational value and clinical implications

The report offers several clinically relevant observations. First, it provides detailed longitudinal documentation of immune profile evolution at two time points (2 and 6 months) in the same infant, revealing a transition from quantitatively normal T-cell counts to profound lymphopenia—a temporal pattern that, to our knowledge, has rarely been documented in such detail in an infant subsequently diagnosed with XHIGM. Second, it illustrates the utility of mNGS on induced sputum (with 34,002 reads) as a rapid, non-invasive diagnostic tool for PJP in a critically ill infant where BAL was not feasible, and demonstrates how high read counts, combined with clinical and radiological findings, can distinguish active infection from colonization. Third, it highlights that normal early T-cell counts and IgM can be falsely reassuring; serial immunological evaluation and early genetic testing should be pursued when clinical clues (recurrent infections, failure to thrive, opportunistic infection) persist.

## Conclusion

This case underscores three key messages. First, declining T-cell counts and persistently low IgA in infants with recurrent or opportunistic infections warrant urgent genetic evaluation, even when initial screening appears normal. Second, opportunistic infections—particularly PJP—or recurrent site-specific infections in infancy should trigger timely genetic testing to enable early diagnosis and transplantation planning. Third, aggressive multidisciplinary management is essential to improve outcomes in this vulnerable population.

## Data Availability

The clinical and laboratory data of the patient are available on our hospital software system. Further inquiries can be directed to the corresponding author.
